# A Robotic Picking Framework for Autonomous Singulation of Sweetpotato Slips into Chainpots

**DOI:** 10.3390/s26206453

**Published:** 2026-10-12

**Authors:** Prem Raj, Weilong He, Alexandria Brown, Yuxi Chen, Azlan Zahid, Lirong Xiang

**Affiliations:** 1 Department of Biological and Agricultural Engineering, North Carolina State University, Raleigh, NC 27606, USA; prem.raj@tamu.edu (P.R.); wh542@cornell.edu (W.H.); albrow22@ncsu.edu (A.B.); ychen289@ncsu.edu (Y.C.); 2 Department of Biological and Agricultural Engineering, Texas A&M AgriLife Research, Texas A&M University, Dallas, TX 75252, USA; azlan.zahid@ag.tamu.edu; 3 Department of Biological & Environmental Engineering, Cornell University, Ithaca, NY 14853, USA

**Keywords:** robotic transplanting, grasp planning, instance segmentation, keypoint detection

## Abstract

Labor shortages in agriculture, particularly for tasks requiring high dexterity such as sweetpotato transplanting, have increased the need for robotic automation. Although automated transplanting machines are available, they require manually singulated slips to be loaded into chainpots, making the overall process labor-intensive and costly. To address this limitation, this work investigates the feasibility and challenges of a robotic framework for automating the slip singulation task in chainpots. The proposed framework integrates a general-purpose robotic manipulator, a 3D camera, and a parallel-jaw gripper. The perception system consists of two core modules: (1) a deep learning-based instance segmentation and keypoint detection model for localizing individual slips and estimating their transplanting direction, and (2) a simple analytical grasp-planning algorithm for computing stable and collision-free grasp poses. To overcome the limited availability of annotated training data, a novel data generation pipeline is developed to create a large synthetic annotated dataset from a small collection of real-world images. The proposed pipeline generates photorealistic cluttered scenes by randomly compositing annotated top-down images of individual slips onto a target background while simultaneously applying domain randomization. During real-world experiments conducted under controlled laboratory conditions, the robotic framework achieved a grasp success rate of 72–100% and a transplanting success rate of 75–88%, depending on the degree of slip overlap, occlusion, and leaf entanglement. The proposed framework represents an initial important step toward the development of a fully automated robotic system for sweetpotato slip transplanting.

## 1. Introduction

The global agricultural sector is experiencing a significant labor shortage, posing major challenges to agricultural productivity and sustainability. The Organisation for Economic Co-operation and Development (OECD) reported that approximately 2.5 million workers left the agricultural sector across the European Union over the last decade [1] This trend is particularly evident in specialty crops such as sweetpotato, where transplanting is a labor-intensive, time-consuming, and costly operation. Previous studies have identified transplanting as a major contributor to production costs and have highlighted the continued reliance on manual labor despite the availability of mechanical transplanting technologies [2,3,4]. Given the persistent labor shortage and the importance of sweetpotato as a staple crop cultivated in over 100 countries [5], automating the transplanting process has become increasingly important.

The transplanting process for sweetpotatoes differs substantially from that of many vegetable crops, such as lettuce, tomato, and cabbage, which are propagated from seeds. In these crops, seedlings are directly grown in chainpots or trays, enabling relatively straightforward automated transplanting [6,7,8,9,10]. In contrast, sweetpotatoes are propagated using stem cuttings, commonly referred to as slips [11]. After harvesting, slips are collected as loosely piled bunches with varying degrees of overlap and leaf entanglement, making automated handling considerably more challenging [12,13,14]. In conventional transplanting, operators manually separate individual slips, identify their planting orientation, and feed them into the transplanter for insertion into the soil [15]. Although several semi-automatic transplanters have been developed to automatically remove seedlings from chainpots and transplant them into the soil [16,17], these systems assume that planting materials are already individually organized. For sweetpotato production, the preparation and handling of individual slips before transplanting remain largely manual. Developing robotic techniques for perception, grasp planning, and manipulation of individual sweetpotato slips therefore represents an important step toward the full automation of the overall transplanting process.

Automating the picking of cluttered sweetpotato slips presents several perception and manipulation challenges [18]. Sweetpotato slips are thin, elongated, and exhibit similar visual appearance, making reliable instance detection difficult, particularly under overlap and occlusion. Grasp execution is further complicated by their slender geometry and fragile structure. Suction grippers are generally unsuitable because they require sufficient contact area to establish an airtight seal [19]. Parallel-jaw grippers provide a more suitable alternative; however, they require accurate grasp pose estimation to avoid grasp failure and collisions with the supporting surface [20]. In addition, estimating the planting orientation of each slip is essential for correct insertion into the chainpot. A mechanical system for sweetpotato slip singulation and transplanting was previously investigated [18]; however, its performance was constrained by limited perception capabilities for reliable slip localization and by the difficulty of manipulating thin, delicate plant material. More recent studies have demonstrated the use of advanced vision-based perception and robotic manipulation for harvesting and sorting agricultural produce, such as tomato trusses [21]. However, sweetpotato slips differ substantially from such bulkier and more rigid crops, requiring specialized perception, grasp planning, and manipulation strategies.

To address these challenges, this study explores a robotic framework for automating sweetpotato slip picking and insertion through a sequential pipeline consisting of perception, grasp planning, grasp execution, and insertion. The perception module performs two key tasks: instance segmentation to localize individual slips and direction estimation to determine their appropriate orientation for insertion.

Instance segmentation is a computer vision task that aims to identify and segment individual object instances at the pixel level [22]. Unlike semantic segmentation, where all pixels belonging to the same object category are assigned a common label, instance segmentation assigns unique labels to different instances of the same object class. Deep learning has become the dominant approach for addressing instance segmentation problems, achieving state-of-the-art performance across various applications [23,24,25,26].

Several deep learning architectures have been developed for instance segmentation, including two-stage convolutional neural network (CNN) models such as Mask R-CNN [25], one-stage CNN models such as YOLACT [27], and transformer-based models such as DETR [28], SAM [29], and SEEM [30]. One-stage models generally provide faster inference but may compromise segmentation accuracy, making them suitable for applications requiring high-speed robotic perception [31]. Transformer-based approaches provide strong generalization capabilities; however, they typically require higher computational resources and exhibit slower inference speeds [32]. Additionally, transformers need massive datasets to learn spatial relationships from scratch, whereas CNNs have built-in spatial awareness (“inductive bias”) that helps them perform exceptionally well on smaller or specialized datasets. Two-stage CNN detectors trained for specific application scenarios generally provide higher segmentation accuracy while maintaining moderate inference speed, offering a favorable balance between accuracy and computational efficiency.

Accurate estimation of the slip planting direction is another critical requirement for successful transplanting. Since sweetpotato slips have an inherent directional structure, with the head and tail regions requiring different placement orientations, identifying their pose is essential for proper insertion into the chainpot. In this study, slip direction estimation is formulated as a keypoint detection problem [33], where the head and tail locations are predicted to determine the overall orientation of each slip. A multitask learning approach is adopted, where a single CNN model shares a common feature extraction backbone while simultaneously predicting instance segmentation masks and keypoints. Such an approach enables joint learning of complementary visual features and provides a computationally efficient solution for simultaneous slip localization and orientation estimation. One question that may arise is why instance segmentation is necessary and why we cannot simply predict keypoints to describe a slip. Segmentation enables the grasp-planning algorithm to reliably pick individual slips while avoiding collisions between the gripper and nearby slips. Without segmentation, the planner cannot determine where the other slips are on the surface. Furthermore, we cannot always assume that the slips will be straight, as they can sometimes be curved. Thus, segmentation is necessary for reliable grasping of sweetpotato slips.

Despite their effectiveness, deep learning-based segmentation models require substantial amounts of annotated data for training, which are often difficult and time-consuming to obtain [34,35]. For sweetpotato slips, manual annotation of segmentation masks is particularly challenging due to their thin structures, similar appearance, and frequent overlap in cluttered arrangements. Therefore, obtaining a sufficiently large high-quality annotated dataset through manual labeling alone is impractical. In this study, a semi-automated data generation strategy is developed to efficiently generate annotated training samples for CNN model training. This approach reduces the dependency on extensive manual annotation while maintaining the required annotation quality. The details of the proposed data generation pipeline are presented in Section 2.2.1.

Using the outputs from the perception module, an analytical grasp planner computes collision-free and stable grasp poses. However, even with accurately estimated grasp locations, grasp execution can fail due to the thin geometry and flexible nature of sweetpotato slips. Furthermore, slips are delicate biological materials that require careful handling to avoid damage. To improve grasp reliability and enable gentle manipulation, a low-cost modification is introduced to the standard parallel-jaw gripper by adding foam padding to the fingertips. The details of this modification are presented in Section 2.1.3.

Similar to sweetpotato, several other crops are propagated through plant cuttings, including cassava [36], yam [37], and taro [38]. Therefore, the proposed robotic framework can potentially be adapted for other cutting-propagated crops with similar handling requirements. However, this study focuses specifically on sweetpotato slip manipulation. Furthermore, the proposed framework can be extended by integrating complementary robotic capabilities, such as branch manipulation [39], toward a fully automated pipeline encompassing slip production, sorting, and transplantation.

The key contributions of this study are summarized as follows:

1. Integration and evaluation of a customized robotic picking pipeline for thin, fragile, visually uniform and cluttered sweetpotato slips.
2. Implementation of a customized visual perception system through efficient synthetic data generation and multitask CNN learning (segmentation and keypoint detection) for simultaneously localizing the slips and determining their planting direction.
3. As a minor contribution, a low-cost fingertip modification for a rigid parallel-jaw gripper is proposed to enable reliable and gentle picking of thin and delicate sweetpotato slips.

## 2. Materials and Methods

**Figure 1:**
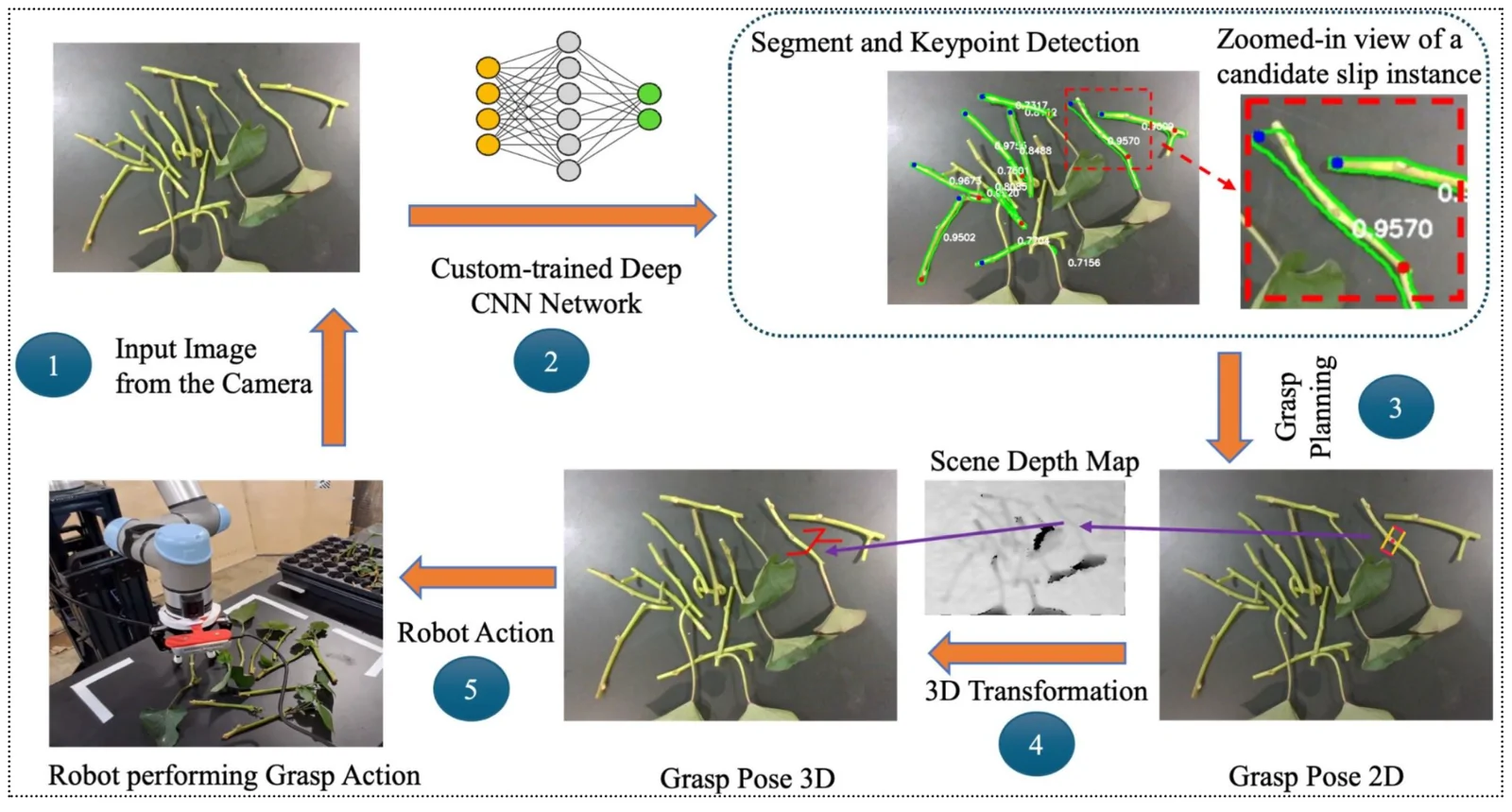
An overview of our slip sorting pipeline. In the first step, the camera captures the input scene with RGB and depth maps. The RGB image is fed to the custom-trained deep CNN, which outputs slip instance segmentations, and directional keypoints (red and blue colored dots on the segmented instances, for head and tail part of slips, respectively). This information is then used as input to the grasp-planning algorithm, which outputs the 2D grasp pose. Using the scene depth map and the camera intrinsic and extrinsic parameters, the grasp pose is transformed into a 3D grasp pose represented in the robot base frame. The resultant grasp pose is sent to the robot to perform the grasping action. Details of each component is described in Section 2.

Our proposed automated slip-sorting framework consists of three main components: a perception module, a grasp-planning module, and a robotic system for grasp execution and slip insertion. An overview of the entire pipeline is presented in Figure 1. Sweetpotato slips are first placed on a flat tabletop, from which the robot aims to pick one slip at a time. The robotic arm observes the scene from a top-down view with the camera mounted on its end-effector. Thereafter, the perception and grasp-planning modules process the visual data and generate an optimal grasp pose. The robot then carries out the grasping and insertion actions. The robotic setup is described in Section 2.1, followed by the perception module in Section 2.2. The grasp planning and execution procedure is detailed in Section 2.3.

### 2.1. Robotic Framework and Setup

In this section, the robotic setup is described, consisting of the robotic manipulator arm along with sensors and tools (Section 2.1.1), the extrinsic calibration process for the robot’s eye-in-hand camera (Section 2.1.2), and the gripper enhancement for handling thin and delicate slips (Section 2.1.3).

#### 2.1.1. Hardware Setup Description

**Figure 2:**
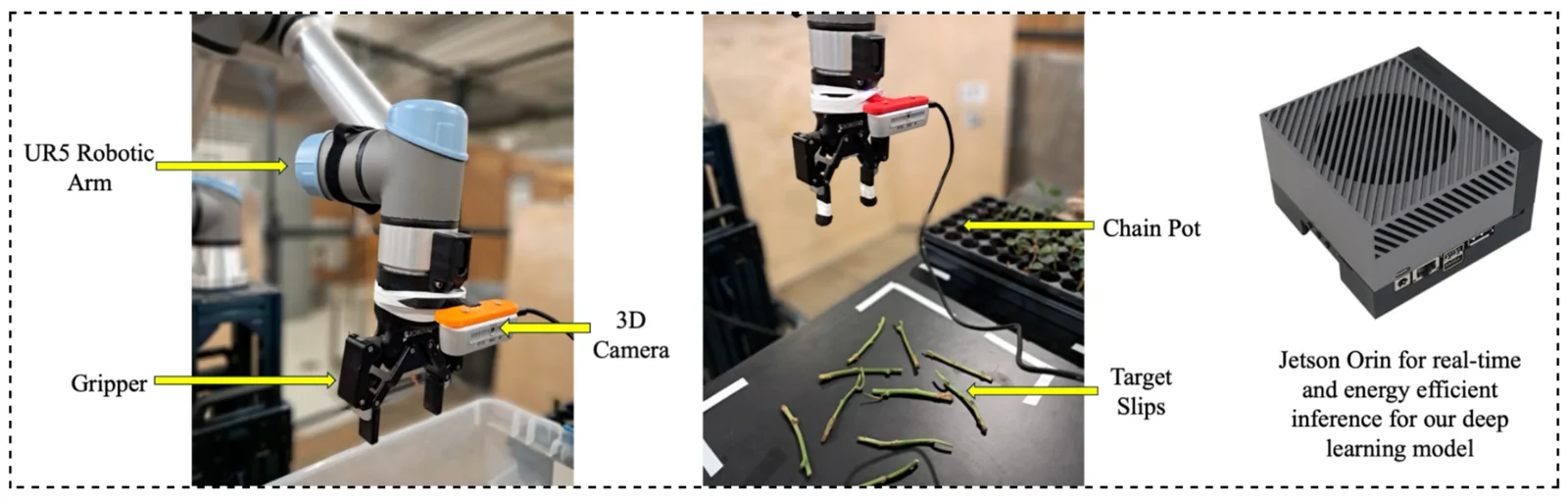
The depiction of the robotic system used for our study. The setup consists of a UR5 robotic arm, a two-finger parallel-jaw gripper, a 3D camera to acquire visual observations, the target slips, the chainpot where slips are inserted in its planting cells after being picked and the Jetson Orin edge device as a processing unit.

The robotic system consists of a UR5e 6-DoF manipulator arm [40], as shown in Figure 2. A 6-DoF industrial manipulator was selected as a general-purpose research platform to investigate the feasibility of perception-guided robotic handling of sweetpotato slips. Although a task-specific low-cost robotic mechanism may provide advantages for large-scale deployment, the objective of this study is to evaluate the fundamental challenges associated with perception, grasp planning, and manipulation of thin and delicate plant materials using a commercially available robotic platform.

The manipulator is equipped with an Intel RealSense D435i RGB-D camera [41], which provides synchronized color and depth information for scene perception. The camera is mounted on the robot end effector in an eye-in-hand configuration, enabling active viewpoint adjustment during the manipulation process. A two-finger parallel-jaw gripper (2F-85, Robotiq, Lévis, QC, Canada) [42] is attached to the end effector for slip grasping. The gripper provides an 85 mm stroke and adjustable force control (20–235 N), allowing controlled interaction with objects of different geometries and stiffness. Since sweetpotato slips are thin and flexible biological materials, additional modifications to improve grasp reliability are introduced and described in Section 2.1.3.

All perception, planning, and robot control computations are performed using an NVIDIA Jetson AGX Orin (NVIDIA Corporation, Santa Clara, CA, USA, 2025) [43] edge AI computing platform. The device integrates an Ampere-based GPU and a 12-core ARM Cortex CPU under a unified memory architecture, enabling efficient processing of vision and robotic control tasks. The Jetson AGX Orin executes the complete slip detection pipeline, grasp planning algorithm, and robot coordination through the Robot Operating System (ROS) [44]. Ubuntu 20.04 is installed on the computing platform, and Python-3.8 is used for perception, planning, and system-level processing.

#### 2.1.2. Robot Hand–Eye Calibration via 3D Point Correspondences

The grasp execution process requires transforming the planned grasp pose from the image coordinate system to the robot reference frame. The transformation from the image plane to the camera coordinate frame is obtained using the pinhole camera model [45], incorporating the camera intrinsic parameters and depth measurements. Subsequently, the transformation between the camera and robot coordinate frames is determined using the camera extrinsic parameters, which define the 6-DoF pose (translation and rotation) of the camera relative to the robot base frame.

**Figure 3:**
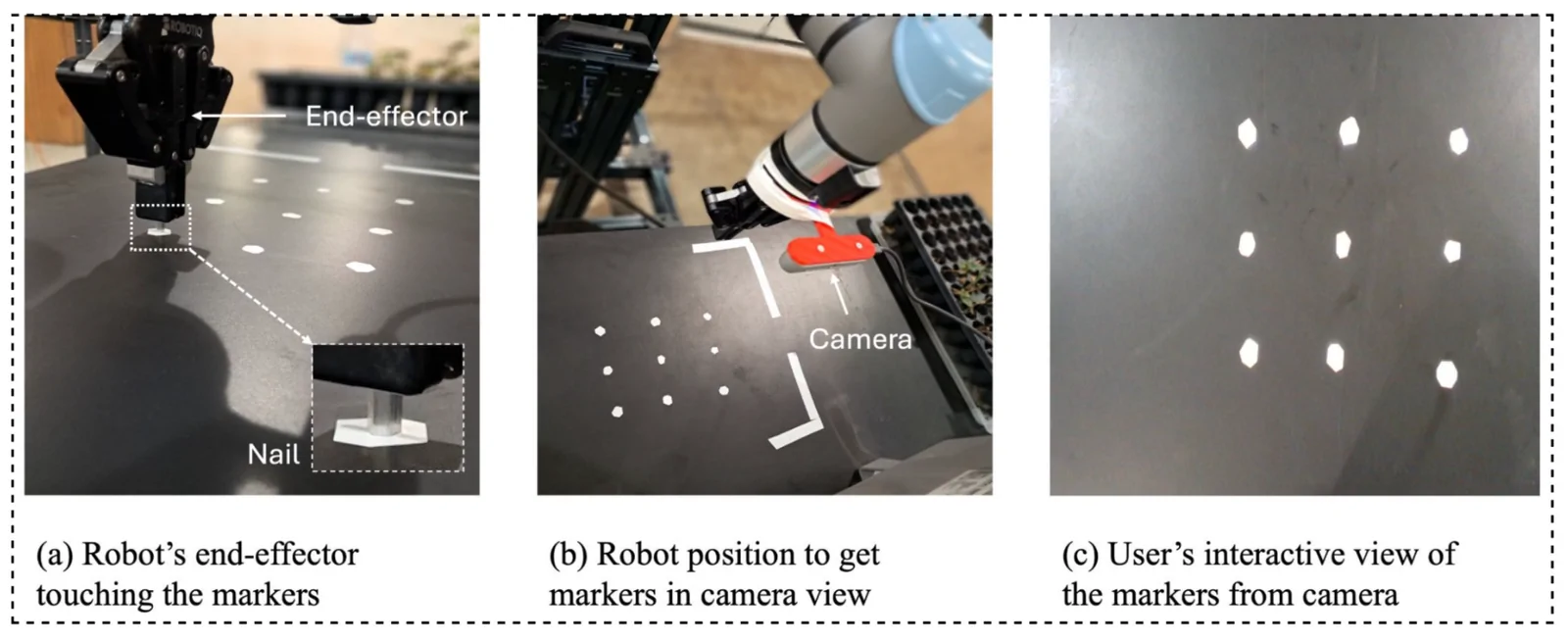
Robot hand–eye camera calibration process. (**a**) The end-effector touches the markers to capture their locations in the robot’s base frame; (**b**) the markers are captured in the camera view, and (**c**) the user clicks on the marker in the same order the robot traversed through them (see Section 2.1.2 for details).

To estimate the camera extrinsic parameters, a hand–eye calibration procedure based on 3D point correspondences is performed. A set of N reference points is placed within the robot workspace (Figure 3a). Since the rigid transformation between two coordinate frames contains six unknown parameters (three translation and three rotation parameters), more than six corresponding points are required. In this study, nine reference points (*N* = 9) are selected to improve robustness against measurement uncertainties from both camera depth sensing and robot positioning.

First, the coordinates of the reference points in the robot coordinate frame are obtained by physically touching each point with the robot end effector and recording the corresponding robot pose from forward kinematics. To provide a well-defined contact location, a pointed tip (nail) is attached between the gripper fingers and used as the end-effector reference point (Figure 3a).

Next, the robot is positioned such that all reference points are within the camera field of view (Figure 3b). The corresponding points in the camera coordinate frame are obtained by manually selecting their 2D pixel locations in the captured image (Figure 3c). The 3D coordinates of these points are then recovered by back-projecting the selected pixels using the measured depth values and the camera intrinsic matrix.

Finally, the rigid transformation between the camera and robot coordinate frames is estimated by solving a point-set registration problem. The optimal rotation and translation parameters that minimize the alignment error between corresponding 3D points are computed using a least-squares solution based on singular value decomposition (SVD). The resulting transformation matrix represents the extrinsic calibration between the eye-in-hand camera and the robot reference frame and is subsequently used for converting vision-based grasp poses into robot motion commands.

#### 2.1.3. Gripper Enhancement for Handling Thin and Delicate Slips

The robotic system employs a commercial Robotiq 2F-85 parallel-jaw gripper. Rather than developing a dedicated soft robotic gripper, the objective of this work is to investigate whether a simple and low-cost modification to a commercially available rigid gripper can improve the reliability of sweetpotato slip manipulation. Although compliant and soft robotic grippers have been extensively investigated for handling delicate agricultural products [46,47], such solutions typically require specialized end-effector designs and additional fabrication. In contrast, this study focuses on a practical retrofit that can be readily integrated into an existing robotic platform.

Sweetpotato slips are slender, delicate, and typically have a stem width of approximately 5 mm. Consequently, successful grasping requires highly accurate positioning of the gripper with respect to the target. If the gripper is positioned even a few millimeters above the desired grasp location, the fingers may fail to establish contact with the slip, resulting in grasp failure. Conversely, if the gripper is positioned slightly lower than intended, the fingertips may collide with the supporting surface, leading to grasp failure and repeated mechanical impacts on the gripper.

**Figure 4:**
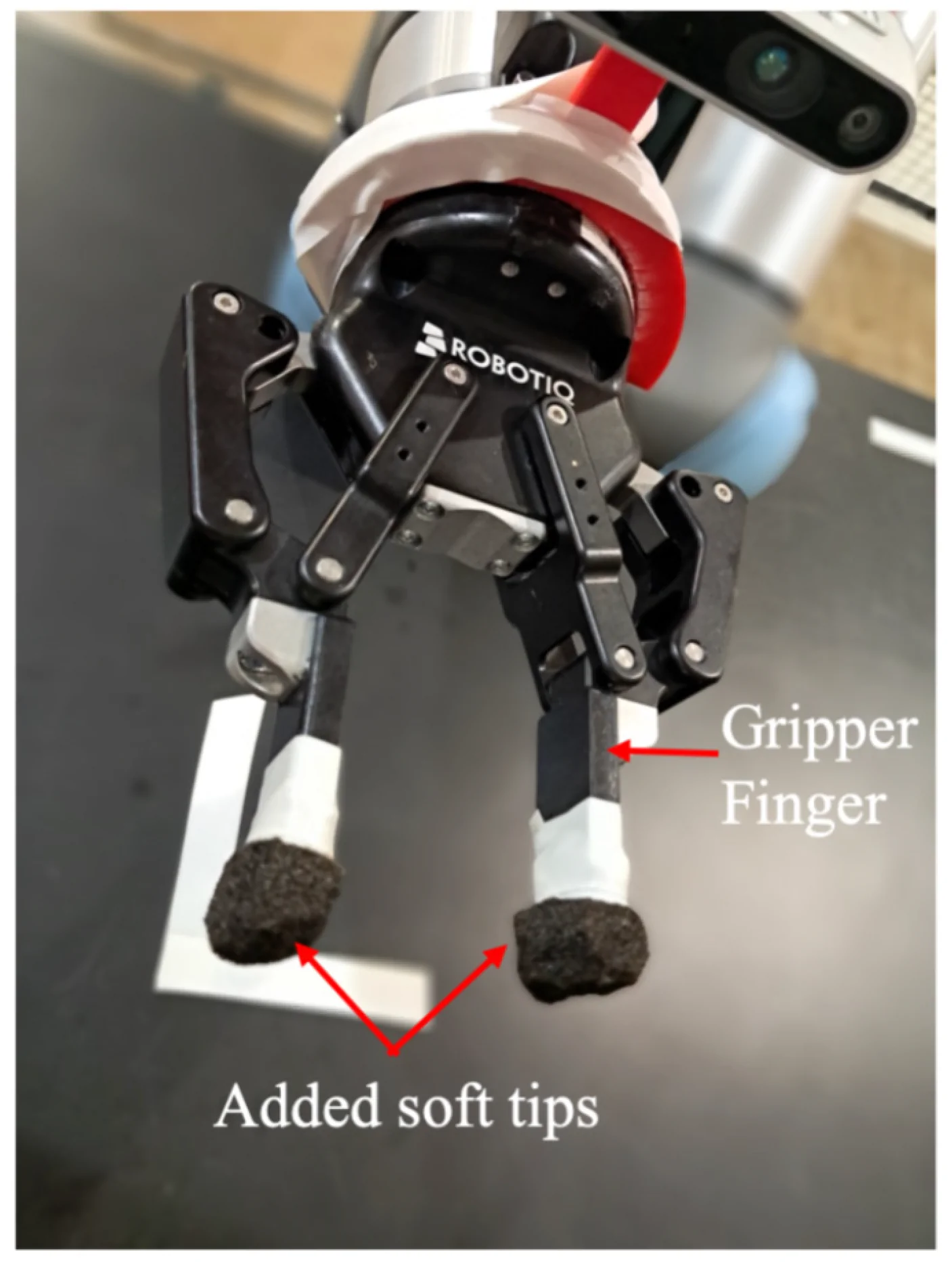
Gripper Enhancement.

To improve robustness against these positioning uncertainties, soft foam padding is attached to the inner surfaces of the gripper fingertips, as shown in Figure 4. The compliant padding absorbs impact when the fingertips contact the table surface, allowing the gripper to tolerate small positioning errors while reducing mechanical stress on the gripper. Furthermore, the compliant contact surface distributes the grasping force more uniformly, thereby reducing the risk of damaging the delicate slips during grasping and transplantation. This simple and inexpensive modification significantly improves the practicality of using a standard rigid parallel-jaw gripper for manipulating thin biological materials without requiring specialized end-effector hardware.

Beyond foam padding, several alternative designs and compliant materials could be considered for handling thin and fragile biological materials. Silicone and rubber elastomers are widely used in compliant robotic grippers because of their stiffness, deformability, and surface properties [47,48]. On the other hand, gel-based materials or elastomeric pads with different hardness levels could potentially distribute contact forces more uniformly. For particularly delicate materials, force or torque sensing could also be incorporated to regulate the applied gripping force. Such material and sensing considerations could provide potential approaches for adapting commercially available rigid grippers to fragile agricultural products and biological materials, with the appropriate design depending on the mechanical properties, geometry, surface characteristics, and manipulation requirements of the target object [49]. A systematic investigation of these alternative considerations is beyond the scope of this work, as the focus of this work is to integrate and evaluate an overall robotic framework.

### 2.2. Perception Module

The perception module is responsible for localizing individual sweetpotato slips and estimating their planting orientation. A deep learning-based perception model is developed for this purpose. Section 2.2.1 describes the proposed training data generation pipeline, while Section 2.2.2 presents the CNN architecture and training procedure.

#### 2.2.1. Training Data Preparation

An efficient data generation strategy is developed to prepare the annotated training dataset required for slip instance segmentation and keypoint detection. Existing approaches for tabletop object segmentation commonly rely on simulation environments to generate synthetic training data [50,51]. However, models trained solely on simulated images often suffer from the domain gap between simulated and real-world environments, leading to reduced performance during real-world deployment [52]. Furthermore, constructing a realistic simulation environment is often labor-intensive.

To address these challenges, the proposed approach directly generates photorealistic synthetic images from a small collection of real-world images. The method exploits two characteristics of the application: (1) sweetpotato slips are thin objects, and (2) the RGB-D camera observes the workspace from an approximately top-down viewpoint. Consequently, the scene can be approximated as planar with only minor height variations.

**Figure 5:**
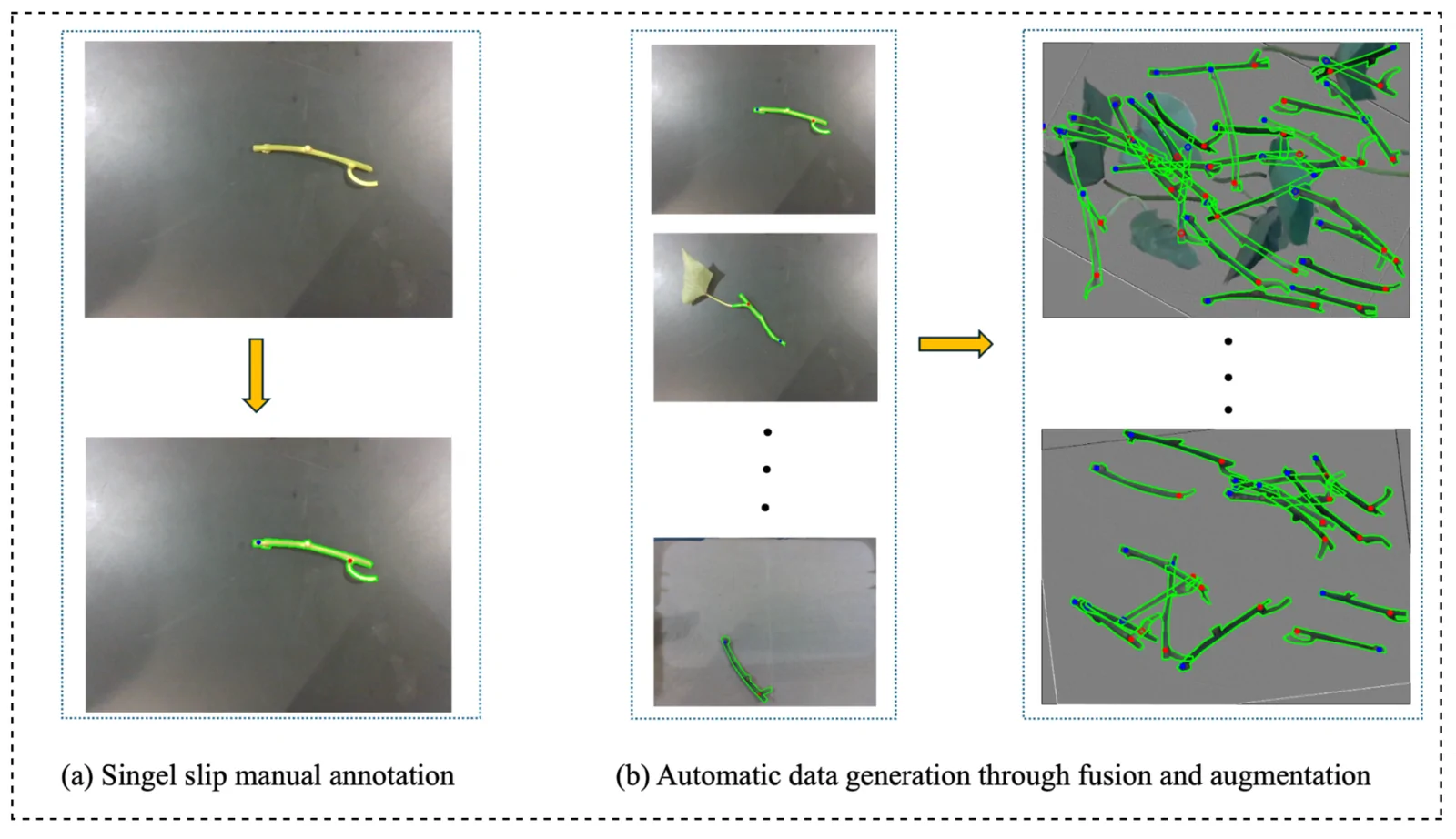
Synthetic training data generation. First, (**a**) a few single-slip images are annotated manually, then (**b**) large real-world training data are generated with our proposed method (Refer to Section 2.2.1 for further details).

First, a small set of top-down images containing a single slip is captured using the robot’s eye-in-hand camera (Figure 5a). These images are manually annotated to obtain the corresponding instance masks and keypoints using the X-Anylabeling software (4.1.0, CVHub, Shenzhen, China) [53]. This process is substantially simpler than annotating cluttered scenes containing multiple overlapping slips. An image of the empty workspace is also captured to serve as the background.

Using the annotated masks, individual slips are automatically extracted from the source images and composited onto the background image using an automated image synthesis pipeline. During image generation, the position of each slip is randomly sampled to create diverse cluttered scenes (Figure 5b). To further improve dataset diversity, domain randomization techniques, including horizontal flipping, random rotation, and color jittering, are applied to both the slips and the background. Since the placement of every synthesized slip is known, the corresponding instance segmentation masks and keypoint annotations are generated automatically without additional manual labeling. The number of slips in each synthesized image is randomly varied between 1 and 30.

The random rotation of the slips is specifically incorporated to balance the distribution of slip orientations within the generated dataset. Since the original top-down images predominantly contain slips aligned in a single planting direction, random rotation produces samples with both upward and downward facing orientations. This improves the directional diversity of the training data and enables the network to learn orientation-invariant features for keypoint detection.

#### 2.2.2. CNN Architecture Design and Training

The perception module requires accurate instance segmentation of individual slips and estimation of their planting direction. Considering the need for both high segmentation accuracy and reasonable inference speed, a two-stage detection framework was selected for this task. Specifically, Mask R-CNN was adopted as the base architecture due to its ability to perform instance segmentation while supporting additional task-specific prediction branches within a shared feature extraction framework.

In addition to instance segmentation, the proposed system estimates the planting direction of each slip through keypoint detection. The direction estimation problem is formulated by identifying two keypoints corresponding to the head and tail regions of the slip. An additional keypoint detection branch is incorporated into the standard Mask R-CNN architecture while sharing the same backbone feature extractor with the segmentation branch. The keypoint branch predicts the spatial location of each keypoint within the corresponding bounding box by generating heatmaps, where the maximum response indicates the predicted keypoint location. Since the proposed framework considers only two classes (i.e., slip and background), the classification layers of the detection head are modified accordingly. Thus, instance segmentation and direction estimation are jointly learned in an end-to-end multitask framework.

The CNN model was trained end-to-end on a workstation equipped with a single NVIDIA RTX A5000 GPU (NVIDIA Corporation, Santa Clara, CA, USA). The Adam optimizer was used with a learning rate of 0.0001, and the model was trained for 30 epochs, requiring approximately 4 h to complete. To improve model generalization and mitigate overfitting, the data augmentation pipeline was expanded to increase the diversity of the training samples, including horizontal flipping, random brightness adjustment, Gaussian noise addition, and affine transformations. In addition, selected backbone layers were frozen during training to restrict model capacity and reduce overfitting. Input images were normalized using the RGB channel-wise mean and standard deviation computed from the training dataset. The generated dataset was divided into training and validation subsets using a 9:1 ratio, where the validation set was used to monitor model performance during training and select the final model. Since the training dataset was photorealistic and representative of real-world conditions, no additional fine-tuning using real-world images was performed. The trained model was subsequently evaluated on an independent real-world test dataset, as described in Section 3.

### 2.3. Grasp Pose Estimation and Execution

Given camera observations, our proposed method integrates machine learning-based perception with analytical model-based reasoning to compute reliable, collision-free grasp poses. The process begins by predicting a planned grasp pose in the image coordinate frame, represented in Equation (1) as follows:

*G_i_* = (*p*, *θ_i_*, *W_i_*, *Q*) (1)

where *p* = (*x*,*y*) is the center of the grasp-pose, *θ_i_* is the grasp-angle with respect to the horizontal axis of the image plane, *W_i_* is the grasp-pose width, and *Q* is the grasp-quality index.

For the robot grasp action, the planner grasp-pose is transformed into the robot reference frame using depth information and camera intrinsic and extrinsic parameters. The resultant 3D grasp-pose *G_r_* in the robot reference frame is represented by Equation (2):

*G_r_* = (*P*,*Θ_r_*,*W_r_*,*Q*) (2)

where *P* = (*x*,*y*,*z*) is the center of the grasp-pose in Cartesian coordinates, *Θ_r_* is the gripper’s rotation around the *z*-axis (top-down), *W_r_* is the gripper’s opening width bounded by its maximum opening, and *Q* is the same as in Equation (1).

For planner grasp-pose prediction, an object-centric framework is employed, consisting of two sequential stages. In the first stage, object-specific features such as slip segmentation masks and orientations are extracted using instance segmentation and keypoint detection. In the second stage, an analytical model-based grasp planning algorithm computes a stable, collision-free grasp pose based on the detected geometry and manipulator constraints.

#### 2.3.1. Grasp Planning

The grasp planning process begins by sorting all detected slip instances according to their segmentation confidence scores in descending order. The confidence score is used only to prioritize the order in which slips are evaluated, as higher-confidence detections generally correspond to more clearly visible and less occluded slips. Each slip is then processed sequentially until a feasible grasp pose is identified.

**Figure 6:**
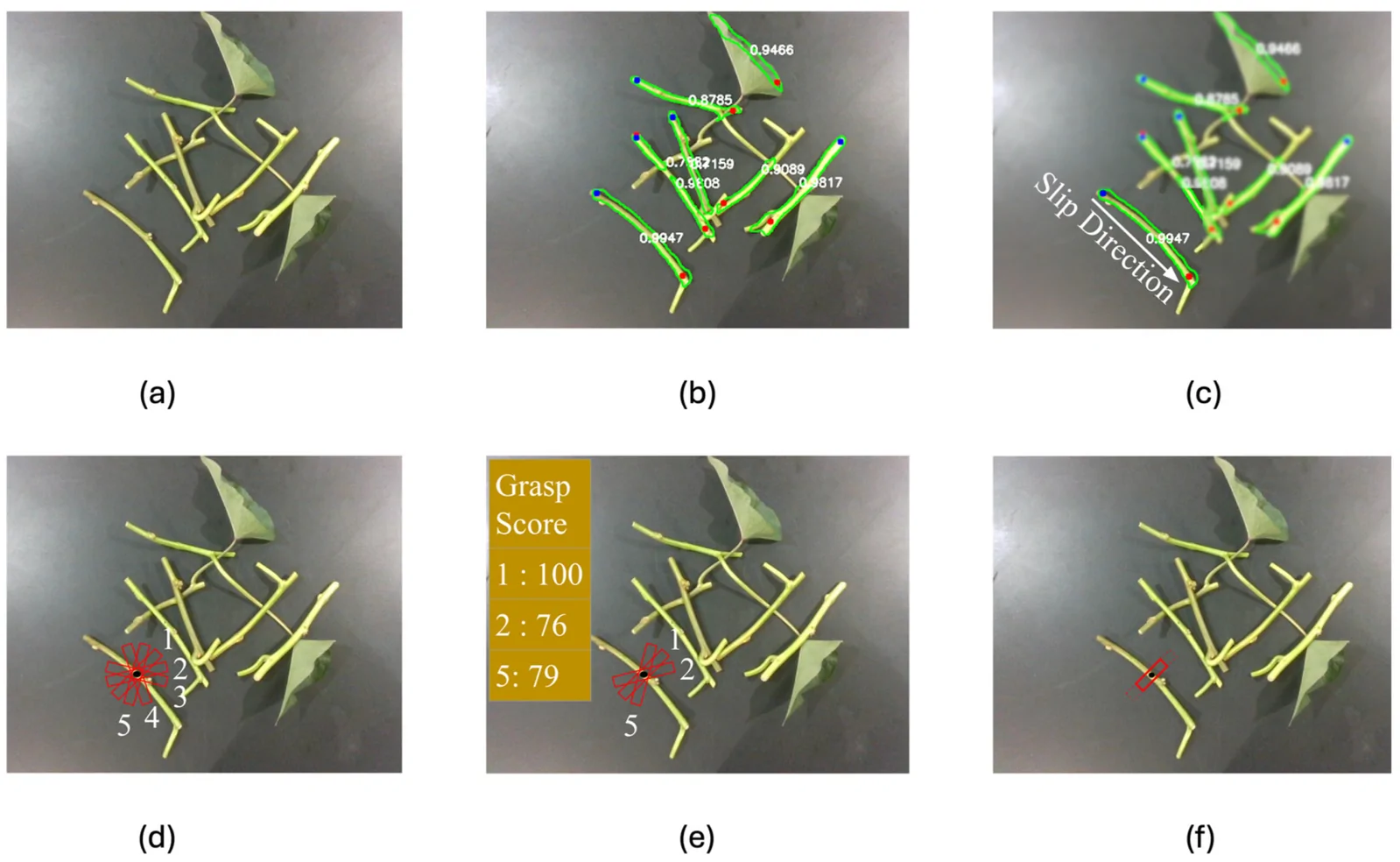
Illustration of the proposed grasp planning pipeline. (**a**) Input RGB image. (**b**) Instance segmentation and keypoint detection results. (**c**) Selection of a detected slip for evaluation and estimation of its planting direction. (**d**) Sampling of candidate grasp poses around the slip center at multiple orientations. (**e**) Analytical grasp pose validation, refinement, and quality scoring. (**f**) Final grasp pose selected for execution. Refer to Section 2.3.1 for details.

For the selected slip instance (Figure 6c), the predicted segmentation mask and keypoints are first evaluated for consistency. Any predicted keypoints lying outside the segmentation mask are discarded. At least one of the two keypoints is required to determine the planting direction. If only one keypoint is available, the missing keypoint is estimated by first computing the major axis of the segmentation mask. The two endpoints of the mask along the major axis are then identified, and the endpoint farthest from the available keypoint is assigned as the missing keypoint. Once both keypoints are available, the planting direction is determined accordingly. In addition, the slip is validated by verifying that its length along the major axis and its width at the midpoint along the minor axis exceed predefined thresholds.

Next, (D = 5) candidate grasp poses are sampled around the center of the slip at orientations of (0, 30, 60, −30 and −60) degrees relative to the minor axis of the segmentation mask (Figure 6d). These candidate poses are evaluated using the analytical grasp planner proposed in our previous work [54]. The planner validates each candidate, refines the grasp pose to improve stability and collision avoidance, and assigns a grasp quality score to quantify its suitability (Figure 6e). Two validity checks are performed: (1) the object width within the grasp rectangle must be smaller than the maximum gripper opening, and (2) sufficient free space must exist around the object to allow collision-free finger insertion. During refinement, the gripper opening is adjusted to improve grasp robustness while accounting for depth sensing errors and robot positioning uncertainties. The candidate with the highest grasp quality score is selected as the final grasp pose for the current slip (Figure 6f).

If no valid grasp pose is found, or if the slip fails the consistency checks, the algorithm proceeds to the next detected slip in descending order of segmentation confidence and repeats the evaluation until a feasible grasp pose is obtained.

#### 2.3.2. Grasp Execution and Overall Flow

The overall robotic framework is implemented using the Robot Operating System (ROS), which manages communication among the perception, planning, and robot control modules. The UR5e manipulator, Robotiq gripper, and Intel RealSense D435i RGB-D camera are interfaced with the NVIDIA Jetson AGX Orin, where all perception, planning, and control computations are performed.

The execution pipeline begins by acquiring synchronized RGB and depth images from the eye-in-hand camera. The RGB image is processed by the CNN to predict instance segmentation masks and keypoints for individual slips. These predictions are passed to the analytical grasp planner, which computes a feasible grasp pose for the selected slip. The predicted grasp pose is subsequently transformed from the camera coordinate frame to the robot base frame using the calibrated hand–eye transformation described in Section 2.1.2. The robot then executes the grasping motion, followed by transplantation of the grasped slip into the designated chainpot.

**Figure 7:**
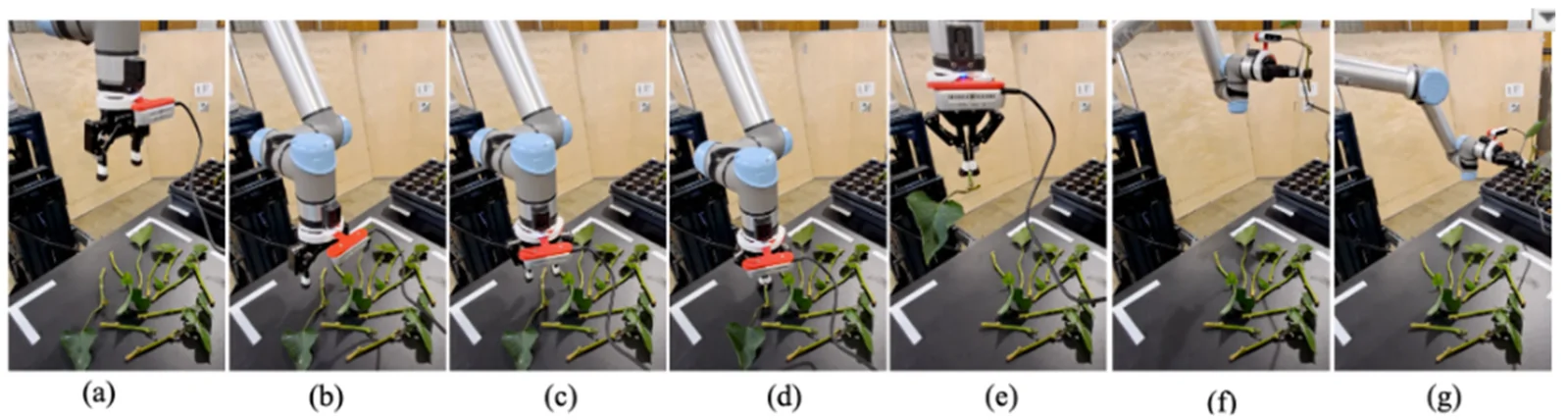
The robotic picking and transplanting pipeline of our proposed system. (**a**) Start position of the robot (**b**) Robot reaching near the target pose (**c**) Gripper orientation and optimized opening of its fingers (**d**) Going down and finger closing for grasping (**e**) Picking the slip (**f**) Intermediate pose above the chainpot (**g**) Transplanting action.

Figure 7 illustrates the complete grasping and transplantation sequence. First, the manipulator moves to an intermediate pose above the target slip. The end effector is then rotated to align with the predicted grasp orientation before descending to the target grasp position. After closing the gripper to secure the slip, the robot retracts to the intermediate pose and subsequently moves to the designated chainpot to complete the transplantation operation.

## 3. Results and Discussion

This section presents the experimental evaluation of the proposed robotic framework. Section 3.1 evaluates the performance of the perception module on the instance segmentation and slip direction estimation tasks under different scenarios. Section 3.2 explains why direction estimation works and how the training data distribution affects it. Section 3.3 presents real-world grasping and transplantation experiments to assess the performance of the complete robotic system. Finally, Section 3.4 presents an ablation study to evaluate the contribution of the proposed gripper enhancement and the influence of hand–eye calibration accuracy on the overall system performance.

### 3.1. Slip Segmentation and Keypoint Detection in Different Scenarios

To evaluate the performance of the trained CNN model for slip instance segmentation and keypoint-based direction estimation, a real-world test dataset was prepared containing diverse slip arrangements. The dataset consists of images captured using the robot’s eye-in-hand camera while slips were placed on the robot workspace table. A total of 255 slip instances distributed across 30 different scenes were included. The test scenes were categorized into three levels of difficulty: Non-overlap, Overlap, and Overlap+Leaves. In the Non-overlap category, slips were placed separately without physical overlap. In the Overlap category, slips were intentionally arranged with partial overlap between neighboring slips. The Overlap+Leaves category represents a more challenging scenario where overlapping slips contain additional leaf structures that introduce further visual ambiguity. All slips used for generating the test dataset were different from those used during training (Section 2.2.1).

The test images were manually annotated using X-Anylabeling software [53]. The trained model was evaluated using two standard instance segmentation metrics: mean intersection over union (mIoU) and mean average precision at an IoU threshold of 0.5 (mAP@0.5), following the COCO evaluation protocol [55]. Predictions with confidence scores below 0.7 were discarded to reduce false positive detections observed at lower confidence thresholds. In addition, a custom direction estimation (DE) accuracy metric was introduced to evaluate the accuracy of slip orientation prediction using keypoints. For all detected slip instances across the test scenes, the predicted head and tail keypoints were used to determine the planting direction. If the direction of M out of N detected slips was correctly estimated, the DE accuracy was calculated as M/N.

**Figure 8:**
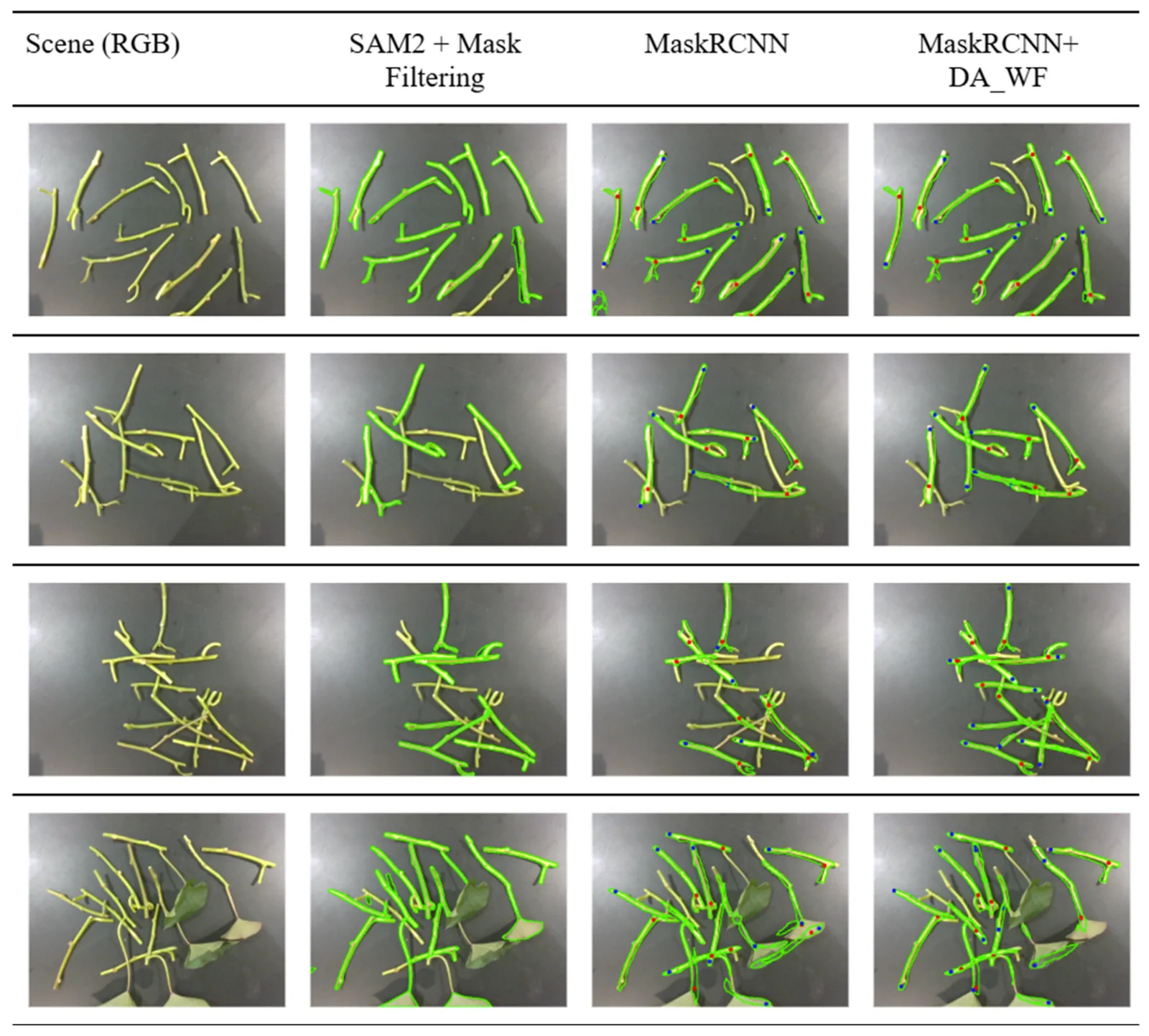
Qualitative results showing the input images (column 1) and the corresponding instance segmentation outputs produced by different models on our custom test set across multiple scene categories. Row 1 contains non-overlap scenes, Rows 2 and 3 show overlap scenes, and Row 4 presents overlap-with-leaves scenes, representing progressively increasing levels of difficulty. Predicted head (red colored) and tail (blue colored) keypoints are also shown for the applicable models. For details, please refer to Section 3.1.

The quantitative results are summarized in Table 1, and qualitative examples from different test scenarios are presented in Figure 8. In addition to the proposed Mask R-CNN-based approach, Segment Anything Model 2 (SAM2) was evaluated as a general-purpose segmentation baseline. Since SAM2 does not provide application-specific slip detection, a mask filtering step was applied to remove segmented regions that did not satisfy predefined slip geometry constraints, such as the minimum width at the mask center along the minor axis. The inference time and segmentation performance of SAM2 were compared with the proposed approach to assess its suitability for robotic manipulation applications.

The results demonstrate that the proposed approach achieves improved performance as the task complexity increases. In non-overlapping scenarios, both SAM2 and the proposed model achieve high segmentation accuracy, indicating that individual slips can be effectively identified when sufficient separation exists between objects. However, the performance of SAM2 decreases in overlapping and overlap-with-leaves scenarios due to the increased visual ambiguity and difficulty in distinguishing individual slip instances. In contrast, the proposed model benefits from task-specific training on synthetic photorealistic slip images and achieves improved mAP@0.5, indicating fewer false positive detections and more reliable instance identification.

Although SAM2 achieves comparable or higher mIoU values in some cases due to its strong boundary localization capability, mAP@0.5 better reflects the suitability of the detection results for robotic manipulation because it penalizes incorrect object instances. The proposed model provides more consistent detections under cluttered conditions, which is critical for downstream grasp planning. Furthermore, SAM2 requires approximately 36.3 s for inference on the NVIDIA edge computing platform (i.e., Jetson AGX Orin), making it unsuitable for real-time robotic operation. In comparison, the proposed model requires only 0.96s per inference, enabling practical integration with the robotic manipulation pipeline.

The purpose of including the Non-overlap category was to establish a baseline performance assessment under a relatively simple condition, where individual slips are clearly separated and less affected by occlusion. Although such arrangements do not fully represent practical transplanting scenarios, they provide a controlled reference for evaluating the impact of increasing scene complexity. In contrast, the Overlap and Overlap+Leaves scenarios progressively introduce more realistic and challenging conditions, where occlusion, similar appearance among neighboring slips, and additional leaf structures increase the difficulty of instance separation. The corresponding reduction in performance across these scenarios highlights the challenges associated with perception in cluttered sweetpotato slip arrangements.

The effect of the proposed training improvements is also evident by comparing the standard Mask R-CNN model with the improved Mask R-CNN+DA_WF model. The latter incorporates an expanded data augmentation strategy and selective backbone layer freezing to increase training diversity and reduce overfitting. The improved model consistently achieves higher segmentation and direction estimation accuracy across all test scenarios, demonstrating the importance of appropriate training strategies for reliable slip perception.

### 3.2. Effect of Random Rotation Augmentation on Direction Estimation

Random rotation augmentation was introduced during the synthetic data generation process to increase the diversity of the training dataset. More importantly, it was designed to address the directional bias present in the original data distribution. Since the original slip images were predominantly captured with a similar orientation, the model could become biased toward predicting a limited range of planting directions. Randomly rotating the synthesized slips enables the generation of diverse head-tail orientations and improves the model’s ability to estimate slip direction.

To evaluate the contribution of random rotation augmentation, the proposed model was trained without this augmentation and compared with the complete training strategy. The direction estimation (DE) accuracy results for the three test scenarios are summarized in Table 1. Without random rotation augmentation, the DE accuracy decreased to 0.76, 0.55, and 0.48 for the Non-overlap, Overlap, and Overlap+Leaves scenarios, respectively. In comparison, the proposed training strategy achieved DE accuracies of 0.95, 0.92, and 0.78 for the corresponding scenarios. These results demonstrate that balancing the orientation distribution during training significantly improves keypoint-based direction estimation, particularly in more challenging cluttered scenarios.

### 3.3. Real-World Transplanting Experiments

To evaluate the performance of the complete robotic framework, real-world slip grasping and transplanting experiments were conducted. For each experiment, slips were randomly placed within the robot’s workspace. The perception module was then used to predict the grasp pose, followed by robotic execution of the grasping and transplantation actions. After each successful or failed trial, the pipeline was repeated until all slips in the current scene were processed. Subsequently, a new scene was generated by randomly placing slips within the workspace for additional grasping trials.

A total of 100 grasp trials were performed for each of the four categories defined in Section 3.1: (1) Without Leaf Non-overlap, (2) Without Leaf Overlap, (3) With Leaf Non-overlap, and (4) With Leaf Overlap. The experimental results are summarized in Table 2. Four evaluation metrics are reported, which are defined as follows:

(1) Grasp Success: A grasp trial is considered successful if the robot successfully grasps a single slip and transports it to the target location (i.e., the chainpot cells).
(2) Multi-pick: A grasp is categorized as a multi-pick if multiple slips are simultaneously grasped during a single trial. Multi-pick cases are considered failed grasp attempts.
(3) Slip Direction Correctness: This metric represents the number of successfully grasped slips that are oriented correctly for transplantation. In multi-pick cases, the direction correctness of the primary target slip is considered.
(4) Transplanting Success: A transplantation is considered successful if the grasped slip is correctly inserted into the designated chainpot cells. If the slip falls out of the chainpot after placement, the trial is considered unsuccessful. In multi-pick cases, only the primary target slip is considered for evaluating transplantation success.

The results in Table 2 show that all grasp failures occurred due to multi-pick events, which were primarily caused by slip overlap and leaf entanglement. Overall, the robotic system was able to reliably grasp the target slip under different clutter conditions. This performance can be attributed in part to the compliant fingertips added to the gripper, which improved tolerance to small positioning errors while enabling stable and damage-free handling of the delicate slips.

Slips without leaves were easier to manipulate compared to slips containing leaf structures. In the With Leaf categories, a higher number of multi-pick cases were observed, mainly due to the entanglement between the target slip and surrounding leaves during grasp execution. Similarly, the Overlap category resulted in reduced grasp success rates due to increased clutter and partial occlusions between neighboring slips. Under these conditions, both the perception module and grasp planning algorithm encounter additional challenges, resulting in reduced overall performance.

The complete grasp prediction pipeline was executed on the embedded NVIDIA Jetson AGX Orin platform. The grasp pose prediction process required approximately 1.02 s, of which approximately 0.96 s was consumed by CNN inference. The complete robotic grasping and transplantation operation required approximately 20 s per slip in the current experimental setup.

### 3.4. Ablation Study

**Figure 9:**
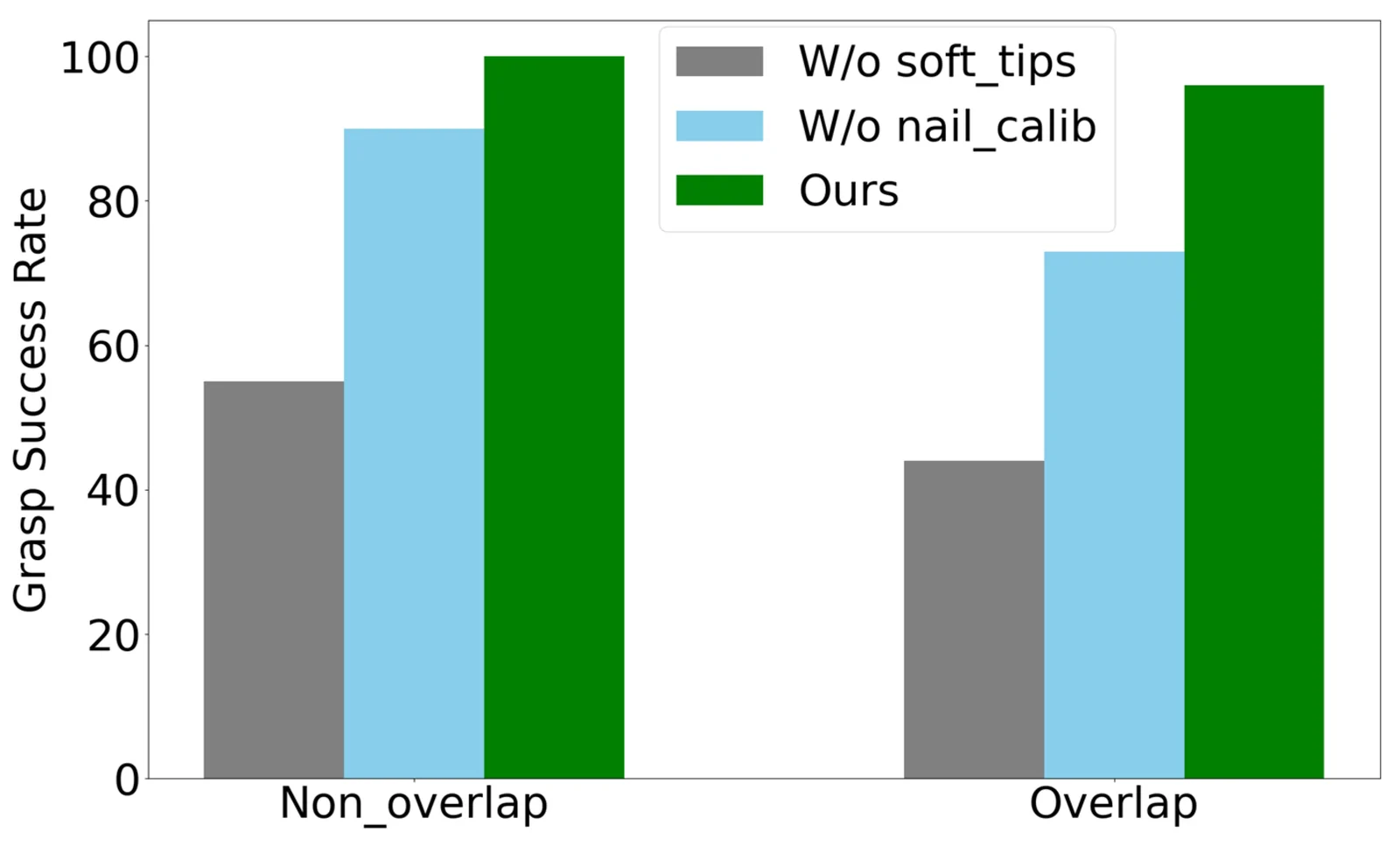
Grasp success rate plot for our full method along with its two ablated versions, tested in two different scenarios, namely ‘Non_overlap’ and ‘Overlap’ for slips without leaves.

An ablation study was conducted to evaluate the contribution of two components in the proposed framework: the soft fingertips added to the gripper and the nail-assisted calibration procedure. The first ablated version, denoted as ‘W/o soft_tips’, was obtained by removing the soft fingertips from the gripper during grasp experiments. The second ablated version, denoted as ‘W/o nail_calib’, used calibration parameters obtained without the nail-based marker localization procedure. For both ablation experiments, all other parameters were kept identical to the proposed framework. A total of 100 grasp trials were performed for each ablated version in two scenarios, namely ‘Non-overlap’ and ‘Overlap’, using slips without leaves as defined in Section 3.1. The results are presented in Figure 9.

For the ‘W/o soft_tips’ configuration, grasp failures primarily occurred due to either missing the target slip or collision between the gripper and the supporting table surface. Due to the small width of the slips, accurate vertical positioning of the gripper relative to the target object is critical for successful grasp execution. However, multiple sources of uncertainty, including calibration errors, depth estimation errors, and robot positioning errors, can accumulate and cause the gripper to be slightly misaligned with respect to the target. In the proposed framework, the addition of soft, deformable fingertips improves tolerance to such positioning errors by allowing safe compression when the fingertips contact the table surface. Consequently, the compliant fingertips increase the allowable vertical positioning error and improve grasp robustness.

For the ‘W/o nail_calib’ configuration, increased calibration errors resulted in inaccurate estimation of the gripper position relative to the target slip, leading to additional grasp failures. This effect became more prominent in the ‘Overlap’ scenario, where reduced spacing between neighboring slips increased the likelihood of multi-pick events. To quantitatively evaluate the contribution of the nail-assisted calibration procedure, the calibration accuracy was further assessed using an independent set of nine workspace markers. The marker positions obtained from the camera coordinate frame using the calibrated transformation were compared with their reference positions measured through direct robot contact. The root mean square (RMS) localization error was 2.8 mm without the nail and 1.5 mm with the nail-based calibration procedure, representing a 46% reduction in localization error. These results demonstrate that precise marker localization during calibration improves camera-to-robot transformation accuracy, which is essential for reliable manipulation of thin slips in cluttered environments.

### 3.5. Limitations and Failure Cases

Although the proposed framework demonstrates the feasibility of robotic sweetpotato slip singulation and transplantation, several limitations remain. One major challenge is accurately estimating the slip-planting direction. This becomes particularly difficult in cluttered scenarios where slips overlap or are partially occluded by leaves. Due to the similar appearance, thin structure, and deformability of the slips, the predicted keypoints can become inaccurate, resulting in incorrect direction estimation. This limitation is reflected in the keypoint-based direction estimation accuracy (Table 1) and the slip direction correctness metric obtained during robotic experiments (Table 2).

Another challenge is the occurrence of multi-pick cases, which are primarily caused by leaf entanglement and inter-slip interactions. In the grasp evaluation, a multi-pick is considered a failure because the objective is to selectively grasp a single target slip. However, during the subsequent transplantation operation, the outcome of a multi-pick can vary. In some cases, the target slip can still be successfully transplanted while additional slips remain attached to the gripper. In contrast, in other cases, the presence of multiple slips prevents successful insertion. Therefore, multi-pick events negatively affect both grasp reliability and overall transplantation performance.

A further challenge is ensuring consistent placement of the grasped slip into the chainpot. Variations in slip shape, slip angle during grasping, and residual entanglement with surrounding plant material can affect the final insertion accuracy.

## 4. Conclusions and Future Works

The proposed robotic framework demonstrates the feasibility of automating sweetpotato slip singulation in chainpots, with promising performance in real-world experiments. The framework particularly addresses the challenges associated with slips being thin, fragile, visually uniform, and prone to entanglement. The study also demonstrates an efficient approach for training the visual perception system in this relatively less explored and challenging scenario. Real-world experiments with varying levels of difficulty further evaluate the performance of the proposed framework and highlight several challenges that remain to be addressed for reliable robotic manipulation of slips and other similar agricultural materials. Future work should focus on improving both perception and manipulation capabilities. A more robust perception framework is needed to improve instance separation and planting direction estimation, particularly under severe occlusion and complex slip arrangements. Incorporating more advanced learning-based approaches that specifically address overlapping cases [56,57,58] could further improve the reliability of slip localization and planting direction estimation in overlapping slip scenarios.

For robotic manipulation, more adaptive grasp planning strategies could be developed to reduce multi-pick events caused by leaf entanglement and tightly cluttered slips. One solution could be to use a decluttering action prior to a picking action [54,59]. For effective slip insertion, a force-feedback-based adaptive strategy could be employed to detect insertion forces and dynamically correct the insertion angle according to the curvature of the slip. Additionally, while a general-purpose 6-DoF robotic manipulator was used in this feasibility study, future work could investigate the design of a dedicated, low-cost robotic platform optimized for sweetpotato transplanting. Since the picking operation is primarily performed from a top-down configuration, a task-specific manipulator with fewer degrees of freedom may be sufficient, potentially reducing system complexity and cost while improving suitability for field deployment. Future work will also include more extensive comparative evaluations, ablation studies, and robustness testing under a broader range of operating conditions to further characterize the generalizability and reliability of the proposed framework.

## Figures and Tables

**Table 1:** Quantitative performance evaluation of the perception module for the sweetpotato instance segmentation task and transplanting direction estimation (via keypoint detection) on our custom-designed real-world test set. For different models, inference run-time (RT) in seconds, instance segmentation accuracy in terms of mIoU (mean intersection over union) and mAP@0.5 (mean average precision with IoU threshold 0.5), and transplanting direction estimation (DE) accuracy are reported. The entries corresponding to the best-performing model are highlighted in bold in the table. For details, please refer to the discussion in Section 3.1.

Model	RT	Non-Overlap			Overlap			Overlap+Leaves		
		mIoU	mAP@0.5	DE Acc.	mIoU	mAP@0.5	DE Acc.	mIoU	mAP@0.5	DE Acc.
SAM2 + Mask Filtering	36.3	**0.76**	0.93	--	**0.48**	0.57	--	**0.39**	0.23	--
MaskRCNN	**0.96**	0.62	0.83	93.6	0.35	0.49	88.5	0.21	0.22	67.4
MaskRCNN+DA_WF (Ours)	**0.96**	0.72	**0.95**	**98.7**	0.42	**0.61**	**92.3**	0.29	**0.36**	**78.2**

**Table 2:** Real-world slip grasping and transplanting results, calculated over 100 grasp trials for each of the categories. For details and further discussion, please refer to Section 3.3.

Evaluation Metrics	Without Leaf		With Leaf	
	Non-Overlap	Overlap	Non-Overlap	Overlap
Grasp Success	100	96	89	72
Multi-pick	0	4	11	28
Slip Direction Correctness	93	78	80	72
Transplanting Success	88	85	82	75

## Data Availability

Link to the source code: https://github.com/ARLabXiang/Sweetpotato_transplanting (accessed on 3 October 2026). The evaluation dataset and the single slip dataset used to generate the dataset are included in the source code itself. A video illustration of our work is available at: https://youtu.be/8nYL8KNUnNY (accessed on 3 October 2026).
